# Quantitative GC–TCD Measurements of Major Flatus Components: A Preliminary Analysis of the Diet Effect

**DOI:** 10.3390/s22030838

**Published:** 2022-01-22

**Authors:** Rafael Freire, Marianela Mego, Luciana Fontes Oliveira, Silvia Mas, Fernando Azpiroz, Santiago Marco, Antonio Pardo

**Affiliations:** 1 Signal and Information Processing for Sensing Systems, Institute for Bioengineering of Catalonia (IBEC), The Barcelona Institute of Science and Technology, Baldiri Reixac 10-12, 08028 Barcelona, Spain; rfreire@ibecbarcelona.eu (R.F.); loliveira@ibecbarcelona.eu (L.F.O.); smas@ibecbarcelona.eu (S.M.); 2 Hospital General de Catalunya, Pedro i Pons, 1, 08190 Sant Cugat del Vallésa, Spain; marianelamego@hotmail.com; 3 Digestive System Research Unit, University Hospital Vall d’Hebron, Centro de Investigación Biomédica en Red de Enfermedades Hepáticas y Digestivas (Ciberehd) Passeig Vall d’Hebron 119-129, 08035 Barcelona, Spain; azpiroz.fernando@gmail.com; 4 Departament de Medicina, Universitat Autònoma de Barcelona, 08193 Bellaterra, Spain; 5 Department of Electronics and Biomedical Engineering, Universitat de Barcelona, Marti i Franqués 1, 08028 Barcelona, Spain; a.pardo@ub.edu

**Keywords:** major flatus gas components, diet effect on flatus, multilevel principal component analysis, rectal gas collection

## Abstract

The impact of diet and digestive disorders in flatus composition remains largely unexplored. This is partially due to the lack of standardized sampling collection methods, and the easy atmospheric contamination. This paper describes a method to quantitatively determine the major gases in flatus and their application in a nutritional intervention. We describe how to direct sample flatus into Tedlar bags, and simultaneous analysis by gas chromatography–thermal conductivity detection (GC–TCD). Results are analyzed by univariate hypothesis testing and by multilevel principal component analysis. The reported methodology allows simultaneous determination of the five major gases with root mean measurement errors of 0.8% for oxygen (O_2_), 0.9% for nitrogen (N_2_), 0.14% for carbon dioxide (CO_2_), 0.11% for methane (CH_4_), and 0.26% for hydrogen (H_2_). The atmospheric contamination was limited to 0.86 (95% CI: [0.7–1.0])% for oxygen and 3.4 (95% CI: [1.4–5.3])% for nitrogen. As an illustration, the method has been successfully applied to measure the response to a nutritional intervention in a reduced crossover study in healthy subjects.

## 1. Introduction

The volume, production, and elimination of intestinal gas are well understood, and it is also known that the composition of the gas evacuated per anus reflects the metabolic activity of intestinal microbiota [1,2,3]. However, despite multiple reports relating the volatilome of feces with several pathologies [4,5,6,7], the composition of intestinal gas in normal conditions is poorly explored. Consequently, there is a lack of knowledge about what is a “healthy flatulence volatilome” and how to assess the importance of the gas components of flatulence as potential biomarkers of microbiota activity in health and disease.

Different reasons explain the insufficient exploration of intestinal gases as biomarkers for diseases. On the one hand, determining a standard healthy pattern of intestinal gas is challenging due to the large differences in the volume and composition of intestinal gases between individuals [8,9,10]. These differences are attributed to the nature of human gut microbiota, which is responsible for much of the production of intestinal gas. Certainly, the human gut microbiota is extremely diverse both within and between individuals. Within individuals, it is known that many hundreds of different bacterial species can be found in the colon and they contain 100 times more genes than their human host [11,12,13], and within individuals, it is also known that a particular microbiota depends on the colonization history of each individual and it is sensitive to a multiplicity of factors as environment, age, diet, lifestyle, climatic conditions, blood group, diseases, or exposure to antibiotics, among others [11,13,14,15].

On the other hand, collecting intestinal gas samples is an additional source of variability. Due to the complex direct access, results differ depending on the sampling method and the point of measure. Under normal conditions, the major intestinal gases are nitrogen (N_2_), oxygen (O_2_), hydrogen (H_2_), carbon dioxide (CO_2_), and methane (CH_4_). While no large population studies have been found, their expected range of variability can be found in Table 1 [16].

Traces of other compounds such as hydrogen sulfide (H_2_S), methanethiol (CH_3_SH), and sulfur compounds, among others [8,17,18] are also found. Nitrogen and oxygen come mostly from swallowed air during food intake, whereas the rest of the gases are mostly generated by bacterial activity in the colon [10,19,20,21]

There exist a variety of methodologies for the quantitative assessment of intestinal gases, each of which has its advantages and drawbacks. However, they yield divergent results, and, as a consequence, there is still a lack of a standard procedure ready for routine clinical application. Image techniques have been used as an indirect method to locate intestinal gas and measure its volume and its movement, [8,22,23] but they are unable to provide information about the chemical composition. Breath analysis has also been used as an indirect way to measure intestinal gases. Its rationale relies on the ability of gases to diffuse to the bloodstream across the gut mucosa and finally be exhaled through the lungs. From the set of intestinal gases, hydrogen and methane are exclusively generated by gut microbiota activity, and they feature a good correlation with their presence in breath [8,24,25]. For this reason, hydrogen- and methane-based breath testing are used in clinical routine for some medical conditions such as carbohydrate maldigestion syndrome or small intestinal bacterial overgrowth [24,26,27]. However, the reproducibility and reliability of the breath test for other intestinal gases are poor, either because of their low concentration in breath or because their origin cannot exclusively be assigned to intestinal activity [21,28].

Several studies have suggested that the VOC profile produced by in vitro incubation of fecal samples can also be used for diagnostic purposes [4,29,30]. These studies rely on the good correspondence between the microbial population of the colon and the microbiota found in the feces. Thus, alterations in VOCs profile of feces should correlate with alterations in the behavior of gut microbial activity due to some gastrointestinal conditions. Although the method has proven its validity in several studies, the VOC profiles measured by in vitro incubation of feces are a simplification of the complex environment of the colon that may not reflect adequately the gas colonic composition because it cannot account for intestinal gas homeostasis, which is a highly dynamic process that depends on the metabolic activity of the intestinal microbiota and the permeability and dynamics of the gas absorption pathways [9,18,31,32]

Despite its invasive nature, direct sampling is likely to provide much more accurate information about the actual composition of the intestinal gases and should perform better in the task of discovering gas biomarkers for intestinal conditions.

The most accessible part of the intestine is the rectum and the usual method for collecting intestinal gases consists of inserting a flexible catheter into it. The reliability in the direct rectal gas sampling method is based on the reported observation that, although the variability is greater, the composition of the collected samples is a good representation of the composition of colonic gas [8,9,18]

However, the direct rectal gas collection is challenging due to the easy contamination of samples with atmospheric gases. Different methods have been proposed to prevent contamination, but there is a lack of a standard procedure on this issue. Actually, a great diversity of protocols is reported that differ in many different aspects of the procedure such as the collecting time, diet, gas wash-out technique, characteristics of the inserting tube, insertion longitude, gas line longitude, sigmoidoscopy assistance, barostat assistance, collecting bag, and infusion of reference gas, among others [10,20,31,33,34,35,36].

An interesting alternative is the use of ingestible gas-sensing capsules that travel along the digestive tract measuring and transmitting the gas composition in real-time. This alternative is less invasive than rectal or colonic catheter insertion but is still immature, and further work is needed before its use in clinical routines [8,28].

An additional alternative could arise from the sensors used at biogas production plants. Biogas is a complex mixture of gases produced by the fermentation of organic matter. Differences aside, a certain correspondence can be established between the anaerobic microbial activity that produces biogas and the bacterial digestive activity that produces intestinal gases. Although in different proportions, biogas contains methane and carbon dioxide as major components. Nitrogen and hydrogen can also be found at low concentrations and oxygen is an undesirable part of the biogas because it reduces its heating power, but it can be also present at trace concentrations [37]. In the biogas industry, it is common to use electrochemical sensors for the measurement of oxygen, thermal conductivity detectors for the measurement of hydrogen, and near-infrared sensors for the measurement of methane and carbon dioxide. Other technologies such as pellistors, metal oxide sensors, and calorimetric sensors, among others, can also be used [38,39]. Several companies have developed instruments integrating different sensor technologies suitable for biogas measurement. Fixed multi-gas analyzer examples are: INCA101 from Union Instruments GmbH, Ei2100 from OhioLumex Co., Inc, and Click! System LTD, among others. Examples of portable multi-gas monitoring systems are Gas Data BlueVary from BlueSens gas sensors GmbH, Biogas5000 from QED environmental systems LTD, and GX3R from RKI Instruments Inc, among others. These devices, or similar ones, are potentially useful for the measurement of major gas intestinal components. Their measurement ranges cover most of the intestinal gas ranges described in Table 1, except for hydrogen, which should be extended. The typical ranges declared by the manufacturers for every gas are methane (0–100%), carbon dioxide (0–100%), and oxygen (0–25%). For hydrogen, the ranges vary, and the larger min–max range we have found is 0–30%. 

As far as we know, the strategy of using arrays of sensors for intestinal gases’ monitoring has been poorly implemented. Some works have proposed arrays of sensors for the measurement of the headspace of fecal samples [40,41,42,43]. Lab assays, testing the sensitivity of different gas sensor technologies to specific intestinal gas components have also been reported [44,45]. Unfortunately, most of the low-cost gas sensors are not suitable for working in anaerobic conditions, and their use for directly measuring intestinal gas is poorly explored.

This study presents a procedure for direct rectal gas collection with preventions for atmospheric contamination, quantitative analysis by GC–TCD, and a multivariate data processing method for the analysis of the five major gas components (H_2_, CH_4_, N_2_, O_2_, and CO_2_). The literature in flatus analysis is scarce and, as far as we know, none of the reported studies have performed a combined analysis of the concentrations of all the five major gases. Results show the capability of the procedure to reject the atmospheric contamination of the samples and the ability of the method to accurately measure the major gases’ composition of the samples and discriminate between diets. Five healthy subjects were submitted to two different diets (high-flatulogenic diet and low-flatulogenic diet) and the corresponding gas intestinal samples were collected in duplicate. Multivariate signal processing allowed a clear interpretation of the major changes in the gas intestinal composition of the samples and a good correlation with diet was established.

## 2. Materials and Methods

### 2.1. Participants 

Five healthy subjects (three women, two men; age range: 25–40 years) participated in the study. The absence of gastrointestinal symptoms related to diseases or disorders was confirmed by a specific clinical questionnaire prior to entry. Antibiotic consumption during the previous two months was an exclusion criterion.

### 2.2. Expèrimental Design

Each subject underwent two studies, at least one week apart, in which intestinal gas production was measured after: (i) 1-day low-flatulogenic diet and a low-flatulogenic test meal, (ii) 1-day high-flatulogenic diet and a high-flatulogenic test meal.

### 2.3. Procedure

#### 2.3.1. Dietary Interventions

Participants consumed their usual diet until the day before each study when they were put on the low- or high-flatulogenic diet. The next morning, participants reported to the laboratory after an overnight fast, and the volume of intestinal gas produced over 4 h was measured after the low- or high-flatulogenic test meal. 

On the high-flatulogenic diet, participants were instructed to eat one portion (250 g) of high-flatulogenic foodstuffs (mixed vegetables for lunch and white beans for dinner) together with egg, meat, fish, and a banana for dessert. The high-flatulogenic test meal consisted of white beans (250 g), 1 banana, and 200 mL water.

On the low-flatulogenic diet, they were instructed to eat one portion of pasta, rice, or lettuce for lunch and soup or lettuce for dinner together with egg, meat, or fish and an apple or a pear for dessert. The low-flatulogenic test meal consisted of 200 mL orange juice and a warm sandwich (58 g white bread with 12 g butter, 38 g ham, and 38 g cheese) freshly cooked on a hot plate (Sandwich Maxi 20, Fagor, Olite, Spain) for 3 min and administered at a standard temperature.

#### 2.3.2. Anal Gas Collection

**Figure 1:**
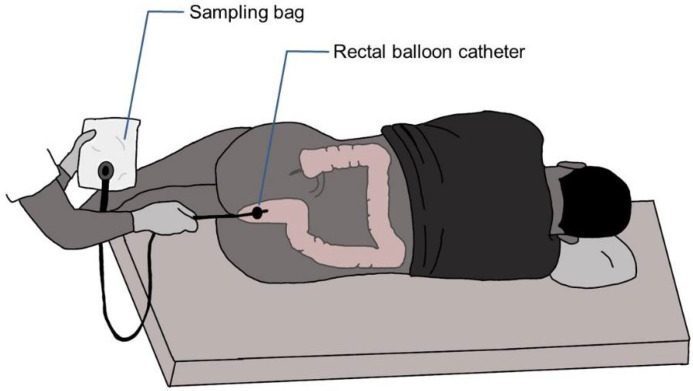
Schema of the rectal gas sample procedure. Rectal gas was collected via a balloon catheter connected directly to a gas collection bag.

During 4 h after ingestion of the test meal, rectal gas was collected via a balloon catheter (20 F Foley catheter, Bard, Barcelona, Spain) connected directly to a special gas collection bag (Restek Sampling Tedlar^®^ bag 7” × 7”, 1 L capacity with polypropylene valve and septum fitting from Restek Co. (Bellefonte, PA, USA), and the volume was measured (see Figure 1). The intrarectal balloon was inflated with 5 mL of water to prevent anal gas leaks. The amounts of hydrogen, carbon dioxide, methane, oxygen, and nitrogen in the gas collected were measured by gas chromatography.

### 2.4. Chromatographic Protocol

The analysis of flatus for major gases was completed using a gas chromatograph Thermo Trace^TM^ 1300 GC equipped with a ShinCarbon ST column (2 m 1 mmID 1/16”OD) and a thermal conductivity detector (TCD Instant connect for Trace^TM^ 1300 GC). The carrier gas used was helium ultra-high purity 6.0 at a flow rate of 15 mL/min and the column pressure was set at 240 KPa. The elution was achieved with a single temperature ramp program: 40 °C for 3 min, 40 to 250 °C at 25 °C/min, and the temperature stayed at 250 °C for the 3 last minutes. The overall time for the measure was 14.4 min. The injector and detector temperatures were at 200 °C and 250 °C, respectively.

Samples, either from standard gases mixture or from real patients, were stored in a 1 L Tedlar sampling bag from Restek^®^. In all cases, no more than 8 h elapsed between gas sampling and gas measurement. Bags have a polypropylene valve suitable for syringe sampling. A 100 µL gas syringe (VICI A-2 series) with Pressure Lok^®^ locker was used for sampling 100 µL of gas from the bag with minimum atmospheric gas contamination. The sample was transferred to the GC column at a flow rate of 100 mL/min. Each bag was measured in duplicate

### 2.5. Standards for the Calibration Curve of the GC Instrument

Two different cylinders (Linde group) of certified standard gas mixtures were used for the calibration of the instrument and validation of the methodology: the first mixture, named A, containing 50% of nitrogen and 50% of hydrogen, and the second mixture, named B, containing 50% nitrogen, 25% carbon dioxide, 20% methane and 5% hydrogen. Mixture A was used to extend the calibration range of hydrogen because for safety reasons its concentration in mixture B cannot be higher. Additionally, for safety reasons, oxygen was not included in the standard mixtures. The calibration for oxygen and nitrogen was carried out by injecting atmospheric air. The calibration ranges in fraction volume percentage for every gas were the following: hydrogen (0–50%), oxygen (0–21%), nitrogen (0–78%), carbon dioxide (0–25%), and methane (0–20%). 

### 2.6. Data Processing

The data analysis was performed in MATLAB R2018b. Chromatograms generated by the GC-TCD instrument were exported as .txt files and loaded into MATLAB. Signal preprocessing (peak boundary detection, baseline correction, and peak alignment) were applied to the chromatograms before any calibration or multivariate procedure.

**Figure 2:**
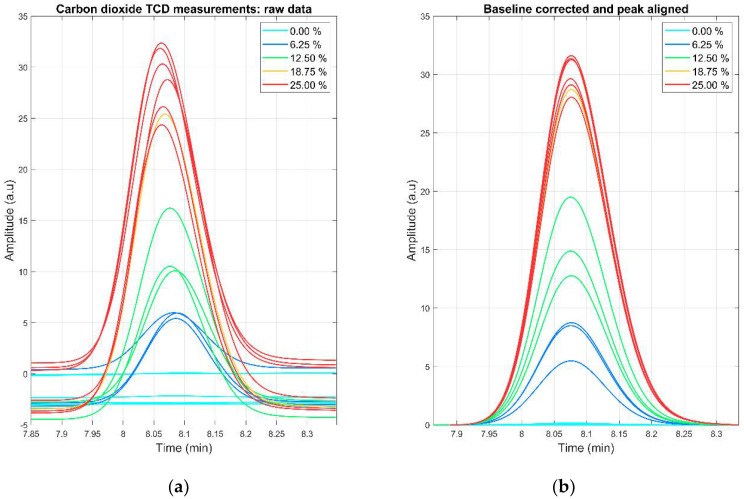
(**a**) Raw carbon dioxide TCD measurements. A misalignment of the peaks and different baseline levels can be observed; (**b**) carbon dioxide TCD measurements after baseline correction and peak alignment.

The baseline for every detected peak was corrected using the AirPLS [46] algorithm and the alignment was performed using the PAFFT algorithm [47]. The peak boundaries were extracted by heuristic inspection. The area of the peaks was calculated using the trapezoidal method and the calibration curve for each day was performed by linear classic least squares. An example of the effects of signal preprocessing in the CO_2_ peak can be observed in Figure 2. The effect for the rest of the analytes can be observed in Figures S1–S4 in the Supplementary Materials. 

For the subject discrimination by diet, multilevel PCA (mPCA)-based k-nearest neighbor (k-NN) analysis was implemented [48]. Alternative multivariate tools such as linear discriminant analysis (LDA) or partial least squares-discriminant analysis (PLS-DA) can be adapted to the multilevel strategy. However, it is well known the tendency of the multivariate supervised algorithms to overfit leads to unreliable scoreplots. Due to the scarcity of observations of our experiment, we preferred to limit the dimensionality reduction to a multilevel PCA. 

Because of the scarcity of data, leave-one-out cross-validation (LOOCV) was used to estimate the performance of the diet classifier.

## 3. Results and Discussion

The calibration of hydrogen, nitrogen, methane, and carbon dioxide was performed by measuring five different dilutions of mixture B in helium. For oxygen calibration, five additional dilutions of air in helium were measured. The dilutions were made directly in the 100 µL gas syringe extracting the gas volumes from two Tedlar bags, one filled with the mixture B (or air) and the other with helium (see Table 2). Extracted volumes followed the numbers shown in the next table. Every dilution was injected into the GC–TCD under the operation conditions previously described.

**Figure 3:**
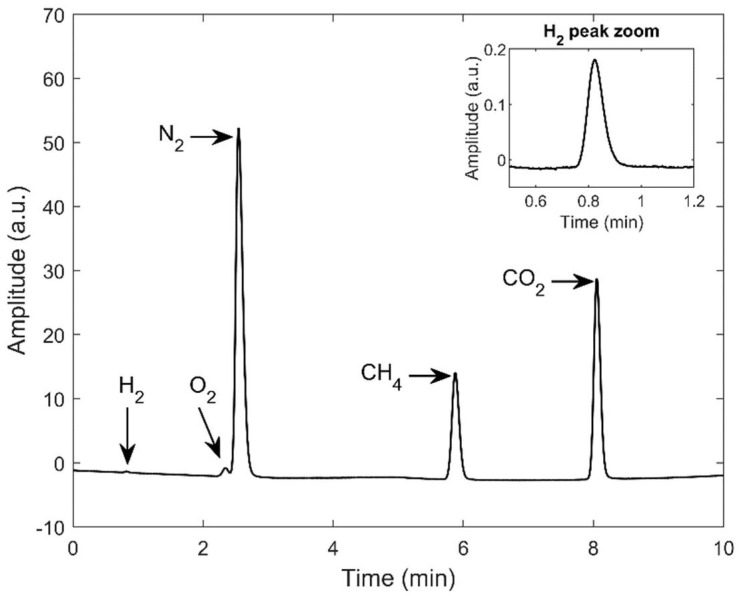
Example of GC–TCD measurement of a sample from the synthetic mixture B.

As can be seen in Figure 3, the gas chromatographic method demonstrated excellent specificity for hydrogen, methane, and carbon dioxide with well-resolved peaks. Oxygen and nitrogen peaks were partially convoluted with a chromatographic resolution of 0.88 showing that no deconvolution algorithms were needed. No other significant peaks were observed for the analysis.

Due to their similar thermal conductivities, the sensibility of the GC–TCD for hydrogen using helium as a carrier gas is very low. However, although the signal is very low, the signal-to-noise ratio is excellent, and calibration is still feasible.

From the measurement of the different dilutions, a model of the calibration curve for every gas was constructed by linear regression. The equation for the calibration line is y = Bx + A, where y is the concentration of the gas measured as a fraction of the volume in percentage over the total volume, B is the slope (in volume fraction/peak area (vf/pa)), x is the area of the chromatographic peak, and A is the intercept (in volume fraction). Values for slope and intercept for every component are shown in Table 3 jointly with their 95% confidence interval in the regression model, the adjusted correlation coefficient (R2), the limit of detection (LOD), and the limit of quantification (LOQ). Calculations of LOD and LOQ are based on the calibration curve according to the following formulas: LOQ = 10s_A_/B, LOD = 3.3s_A_/B, where s_A_ is the standard deviation of the residuals and B is the slope of the calibration curve.

From the inspection of Table 3, we can observe that the sensitivity of the method is maxima for carbon dioxide and minima for hydrogen. From the inspection of the intercept, we can see that we have some contamination with atmospheric air since the concentration of nitrogen at the intercepts is approximately four times that of the oxygen. We can see that we do not have any relevant intercept for methane and carbon dioxide. On the other hand, the intercept for hydrogen can be related to baseline estimation problems since the amplitude of the hydrogen peak is the smallest. From Table 3 we see that the sensitivity for hydrogen is about 50 times smaller than the sensitivity for the other gases. Concerning the limit of detection, we obtain excellent results for all the gases, except for nitrogen, where we obtain a LOD of 3%, also linked to a bigger uncertainty in the intercept. We attribute this to the bigger impact of atmospheric contamination in the case of nitrogen.

### 3.1. Air Contamination

The collection, manipulation, and measurement of either synthetic calibrated gas samples or real patient samples are prone to atmospheric air contamination at every stage of the procedure. To test the robustness of the calibration procedure against atmospheric gas contamination, a helium-filled bag was used to generate twenty 100% helium samples that were injected into the GC–TCD instrument. The peak area for the oxygen and nitrogen was measured and its concentration was estimated using the calibration parameters described in Table 3. The results showed that the mean concentration ± standard deviation for oxygen and nitrogen in samples with 100% helium were 0.84% ± 0.30% and 3.58% ± 0.99%, respectively. These absolute values for contamination are similar to the y-intercept value found for both gases, proving that the procedure is robust to air contamination at the limit of detection level.

### 3.2. Univariate Analysis According to Diet

The total volume of intestinal gas generated by a certain diet fluctuates widely among persons, but it is also well established that a diet rich in fibre causes a significant increase in flatus production in each individual [34,49]. The range of total volume of flatus collected per individual in four hours due to the high-flatulogenic diet was from 43 to 780 mL (median 120 mL), while the range due to the low-flatulogenic diet went from 34 to 439 mL (median 70.5 mL) (See Table S1 for detailed results across participants) These results agree with previous findings. 

For a statistical measure of differences among diets, the paired structure of the experimental design should be considered and, as a first approach, a paired Wilcoxon univariate strategy was implemented. This approach was used to test differences in the volume expelled by subjects under the high- and low-flatulogenic diets. The result of the test determined a statistically significant difference at a 0.05 significance level with a zero median Wilcoxon signed-rank test for paired samples (*p* = 0.03). The column of Total Volume of Figure 1a displays the variability among volunteers and shows the evidence that every individual expels more volume when submitted to the high-flatulogenic diet.

For the measure of major gases, a volume of 100 µL of the collected volume was extracted from the bags with a 100 µL Pressure Lok^®^ glass syringe and introduced in the GC–TCD under the experimental conditions described in the Materials and Methods Section 2.4. Two measurements were performed for each bag: one for estimating the models (calibration dataset) and one for testing the performance of the models (test dataset). With the total amount of volume and the calibration models previously reported the specific volumes of the five major gases were estimated.

The five gases are detected in all measurements: hydrogen, oxygen, nitrogen, methane, and carbon dioxide (for detailed results please consult Table S1 in Supplementary Materials). The predominant contributions to the total volume were from nitrogen, methane, and carbon dioxide, but variability intra individuals was high. The volume of oxygen detected was low but significant and cannot be assigned to atmospheric contamination of samples. The volume of hydrogen detected was very low or even undetectable in some of the samples. This disagrees with the results of Suarez et al. [18] that reported significantly higher concentrations of hydrogen and significantly lower concentrations of methane, but it is in better agreement with the review of Modesto et al. [16]. Considering the large population variability, the different diets, and the reduced number of participants in the reported studies, these differences are not a major concern. 

**Figure 4:**
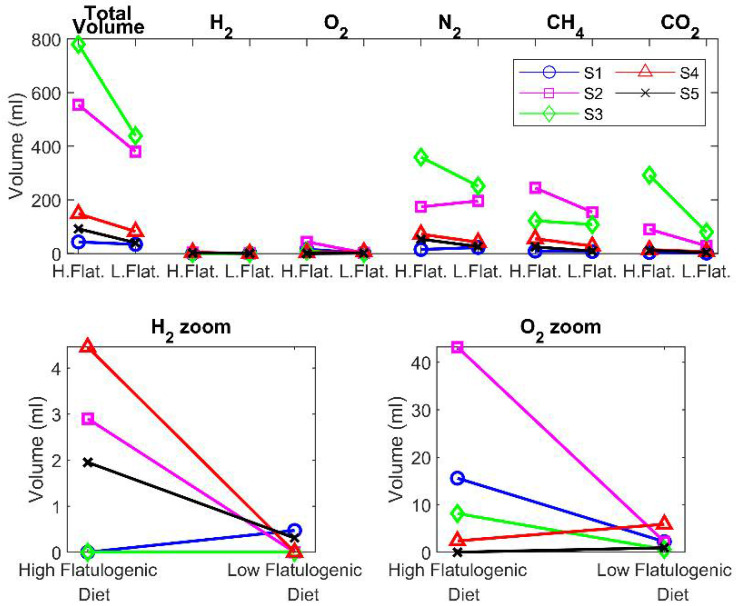
Univariate comparison of diets. Comparison of the total volume of all gases, volume of hydrogen, oxygen, nitrogen, methane, and carbon dioxide produced by subjects submitted to a high-flatulogenic diet and low-flatulogenic diet. S1 to S5 are the subjects.

Figure 4 shows the variability among subjects for the different gases. While the total volume is bigger in the high-flatulogenic diet, this result does not translate automatically to each of the individual gases. Following the same strategy used with the volume, to find significant differences among gases expelled by the high- or low-flatulogenic diet, a paired Wilcoxon signed test was performed. However, all the estimated *p*-values returned by the test were higher than the significance level, which indicates that from the univariate signal processing of each gas, it is not possible to differentiate among diets. This lack of significance may be due to the limited number of participants in the study.

### 3.3. Multivariate Analysis on the Gas Composition of Flatus According to Diet

After the inability of the paired univariate data processing strategy to discriminate among diets, a multivariate strategy was tested. However, since the default multivariate tools ignore the underlying paired structure of the observations, the use of the paired principle had to be adapted to the multivariate data analysis. Multilevel data analysis is an extension of the paired univariate test that allows using multivariate tools for the study of the between-subjects and within-subjects variations in crossover studies [50]. In this paper, we propose the multilevel extensions of PCA as an alternative to their single-level equivalents, which do not consider the crossover experimental design, and to the paired univariate strategy, which only examines one variable per analysis. In our study, multilevel PCA was implemented on the estimation dataset for data inspection and modeling, and a confusion matrix was generated using a k-nearest neighbor’s classifier on the test scores obtained from the test dataset applied and the multilevel PCA model. 

The initial step in the multilevel approximation was the arrangement of the volume observations into a paired data structure to separate the between-subject variation (due to the subjects’ biological intrinsic variability) from the within-subject variation (due to the effect of diet). For the two-class problems we were facing, matrix **F** corresponded to the volume observations of the individual submitted to the high-flatulogenic diet, and matrix **P** corresponded to the volume observations of the same individuals submitted to the low-flatulogenic diet. The paired data arrangement for the between-subject term consisted of the mean matrix (**M**) for all the individuals concatenated in an $\left[\begin{array}{c} M\\ M \end{array}\right]$ arrangement. The paired data arrangement for the within-subject term included the net variation around the mean for all the individuals. The matrix structure of the within arrangement was $\left[\begin{array}{c} -D\\ D \end{array}\right]$ where **D** = [**F** − **P**]. Deeper details about the extension to paired multivariate data analysis can be found in Westerhuis et al. [48]

**Figure 5:**
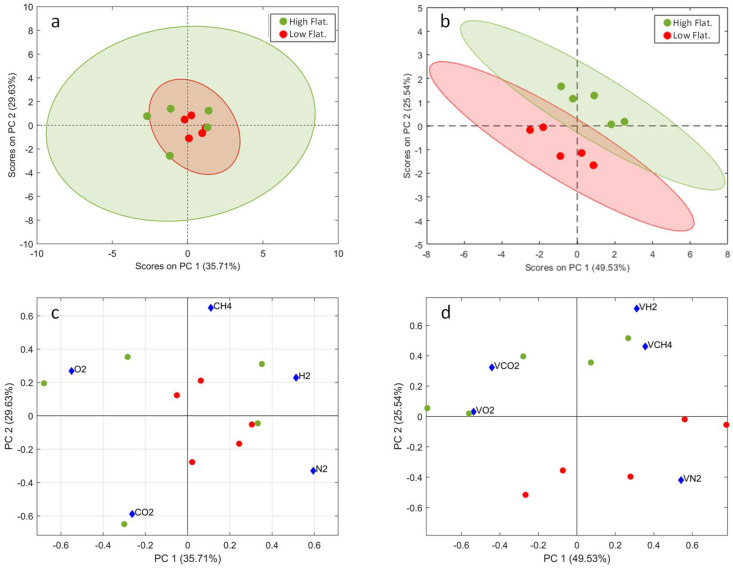
Principal component analysis of the diet data. (**a**) Single-level PCA analysis does not use the paired structure of the experiment, and it is not able to separate between the two diets. (**b**) Multilevel PCS analysis includes the paired structure. Figure shows the PCA of the within-subject variation. A clear separation between classes can be observed. (**c**) A biplot of the scores and loadings of the single-level PCA. No clear association between gases and diet can be extracted. (**d**) The biplot of the multilevel PCA. Nitrogen is close to the low-flatulogenic diet, while methane and hydrogen are closer to the high-flatulogenic diet. Note the PC1 inversion in the comparison between b and d due to the sign ambiguity in the eigenvector.

Next, Figure 5 shows the results of the single-level PCA applied on the autoscaled measured gases dataset and the multilevel PCA applied on the autoscaled within-subject dataset. Single-level PCA was not able to separate the observations by diet, but the multilevel PCA showed well-separated clusters of the two diets. The scatter plots have 95% confidence ellipses drawn on them. The two-dimensional scoreplots contain about 75% of the total paired variance.

The biplot shows a clear correlation between the concentrations of hydrogen and methane, and an anticorrelation between the concentrations of nitrogen on one hand, and carbon dioxide and oxygen on the other. Within the limits of the study, the higher flatulence diet leads to an increased concentration of carbon dioxide coupled with a reduction in the nitrogen content. 

A k-nearest neighbors’ classifier with k = 3 was applied on both the single-level PCA and the multilevel PCA. To test the model performance in predicting the diet, the duplicate set of observations was used. As can be seen in the confusion matrix presented in Table 4, predictions about the diet are perfect in the K-NN applied over the multilevel PCA.

### 3.4. Limitations of the Study and Future Research

The scarce number of participants is a significant limitation of this study. However, it should be noted that the analysis of gases according to diet has been used as a proof of concept of a methodology for sampling and measuring flatus major gas components. It should also be noted that the data analysis and validation have been adapted to the reduced number of samples, avoiding the use of techniques prone to overfitting. Therefore, the authors believe that the resemblance of the estimating and testing datasets as well as the coherence of the observations and consistency of the results concede validity to the implemented methodology. However, the authors agree that a future study with a bigger dataset is needed to confirm the results of this preliminary study.

## 4. Conclusions

Although the knowledge of intestinal gas composition may help to understand the interaction between food intake, the microbiome, and the overall health status, and may serve to develop biomarkers of disease, the study of the concentration profiles of intestinal gases has traditionally received little attention. This insufficient attention can be understood, among other reasons, by the difficulties in the intestinal gas measurements. On one hand, the non-invasive or minimally invasive sampling techniques are not very representative of the actual intestinal gas composition. On the other hand, invasive sampling techniques are complex, they suffer from a lack of standardization, which hinders comparative studies, and they are prone to practical problems such as easy contamination of samples with atmospheric gases. Additionally, metabolic activity in the intestine is a complex process that generates highly dynamic intestinal gas compositions.

The methodology described in this paper can quantify all five major gases (H_2_, CH_4_, N_2_, O_2_, and CO_2_) in a single chromatographic analysis with sufficient accuracy and limit of detection. To avoid atmospheric contamination, samples have been collected using a rectal balloon catheter connected directly to a collection bag especially for gases. Through the calibration models and specific procedures, it has been possible to quantify the mean atmospheric contamination and their variability.

As an illustration of the proposed methodology, the flatus of five volunteers has been analyzed after a nutritional intervention consisting of high- and low-flatulogenic diets. A clear increase in the total expelled volume has been observed for the flatulogenic condition. However, the evolution of single gases is not so clear at the univariate level and also due to the limited sample size of the study. Due to the paired structure of the diet experiment, an extension to multilevel PCA analysis has been used to explore the influence of the diets. The paired extension implementation is simple and separates the between-subject variation (due to the subject’s biological intrinsic variability) from the within-subject variation (due to the effect of diet). Multilevel PCA with projection to two dimensions allowed a perfect class separation that the usual single-level PCA analysis did not achieve. From the inspection of the PCA biplot, it can be concluded that the concentrations of hydrogen and methane were correlated. The concentration of nitrogen was anticorrelated with the concentration of oxygen and carbon dioxide. Nitrogen concentration was in general lower under the high-flatulogenic conditions. 

In summary, this paper describes a methodology for the collection and quantitative analysis of major gases in flatulencies in a single chromatographic analysis. The method has been applied to a nutritional intervention. However, the conclusions related to the latter are only preliminary due to the very limited number of participants in the study. 

## Figures and Tables

**Table 1:** Previously reported concentration ranges in flatus [16].

Gas	Mean ± Standard Deviation (%)	Range (Min–Max) (%)
Nitrogen	65 ± 21	26.0–88.0
Oxygen	2.3 ± 1.0	0–20.0
Hydrogen	2.9 ± 0.7	0.2–49.0
Carbon Dioxide	9.9 ± 1.6	0.7–27.0
Methane	14.4 ± 3.7	0–30.3

**Table 2:** Table of used dilutions of the mixture B in Helium.

	Mixture B (or Air)	Helium
Dilution 1	100% (100 μL)	0% (0 μL)
Dilution 2	75% (75 μL)	25% (25 μL)
Dilution 3	50% (50 μL)	50% (50 μL)
Dilution 4	25% (25 μL)	75% (75 μL)
Dilution 5	0% (0 μL)	100% (100 μL)

**Table 3:** Linear regression parameters for every gas. The table includes estimated values with a 95% interval of confidence. It also shows the R2 of the regression and the limit of detection and limit of quantification for each gas.

Gases	B (%/pa)	Confidence 95%	A (vf)%	Confidence 95%	R2 Adjusted	LOD (%)	LOQ (%)
H_2_	0.94	(−0.91, 0.97)	−0.47	(−1.00, 0.06)	0.98	0.86	2.8
O_2_	0.017	(−0.016, 0.017)	−0.86	(−1.0, −0.7)	0.99	0.24	0.74
N_2_	0.016	(0.015, 0.017)	−3.4	(−5.3, −1.4)	0.96	3.0	9.2
CH_4_	0.020	(0.020, 0.021)	0.0	(−0.2, 0.2)	0.99	0.37	1.1
CO_2_	0.015	(0.014, 0.015)	0.0	(−0.3, 0.3)	0.99	0.49	1.5

**Table 4:** Confusion matrix built on the multilevel PCA analysis.

k-NN over the Multilevel PCA		Actual	
		High Flatulogenic	Low Flatulogenic
Predicted	High Flatulogenic	5	0
	Low Flatulogenic	0	5

## Data Availability

All data supporting reported results are condensed in the table and figures uploaded at the Supplementary Materials.

## Supplementary Materials

The following supporting information can be downloaded at: https://www.mdpi.com/article/10.3390/s22030838/s1, Figure S1: Baseline correction and alignment for hydrogen TCD measurement. The raw data present a clear misalignment of the peaks and differences in the baseline. After correction, all peaks show good alignment, the baseline is addressed, and a clear correlation between peak amplitude and concentration can be noticed. Figure S2: Baseline correction and alignment for oxygen TCD measurement. Again, the raw data present a clear misalignment of the peaks and differences in the baseline. After correction, all peaks show good alignment, the baseline is corrected and a clear correlation between peak amplitude and concentration can be noticed. Figure S3: Baseline correction and alignment for nitrogen TCD measurement. As in the previous cases, the raw data present a clear misalignment of the peaks and differences in the baseline. After correction, all peaks show good alignment, the baseline is addressed, and a clear correlation between peak amplitude and concentration can be better seen. Figure S4: Baseline correction and alignment for methane TCD measurement. Just like previous examples, the raw data present a clear misalignment of the peaks and differences in the baseline. After correction, all peaks show good alignment, the baseline is addressed and a clear correlation between peak amplitude and concentration can be perceived. Table S1: Condensation of the measurement of major gases’ flatus composition of healthy subjects submitted to a diet intervention. Data include 10 measurements and one replicate with the estimated concentration in volume fraction (%) for every gas, jointly with their 95% confidence interval. Table also includes biometric data such as sex, age, and IMC.
