# Supplementary material for: Quantitative GC–TCD Measurements of Major Flatus Components: A Preliminary Analysis of the Diet Effect
Source: Sensors (Basel). 2022 Jan 22;22(3):838. doi: 10.3390/s22030838 (PMC8840200; doi:10.3390/s22030838)
Supplement: Supplementary file 1 [file sensors-22-00838-s001.zip › sensors-1546263-supplementary.pdf]

Supplementary Material

Figure S1: Baseline correction and alignment for Hydrogen TCD measurement

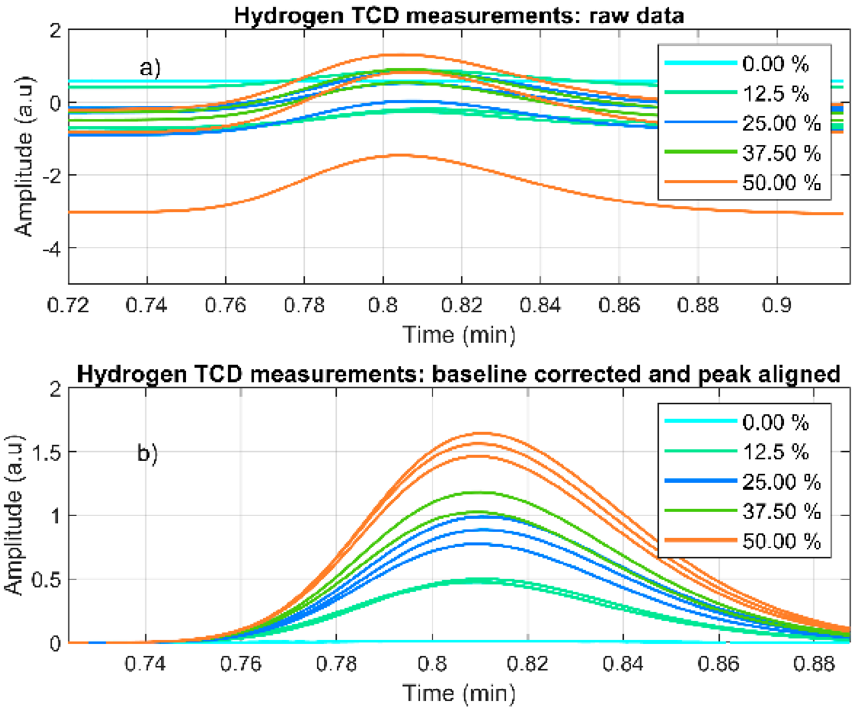

(a) Raw Hydrogen TCD measurements. A misalignment of the peaks and different baseline levels can be observed.

(b) Hydrogen TCD measurements after baseline correction and peak alignment

Figure S2: Baseline correction and alignment for Oxygen TCD measurement

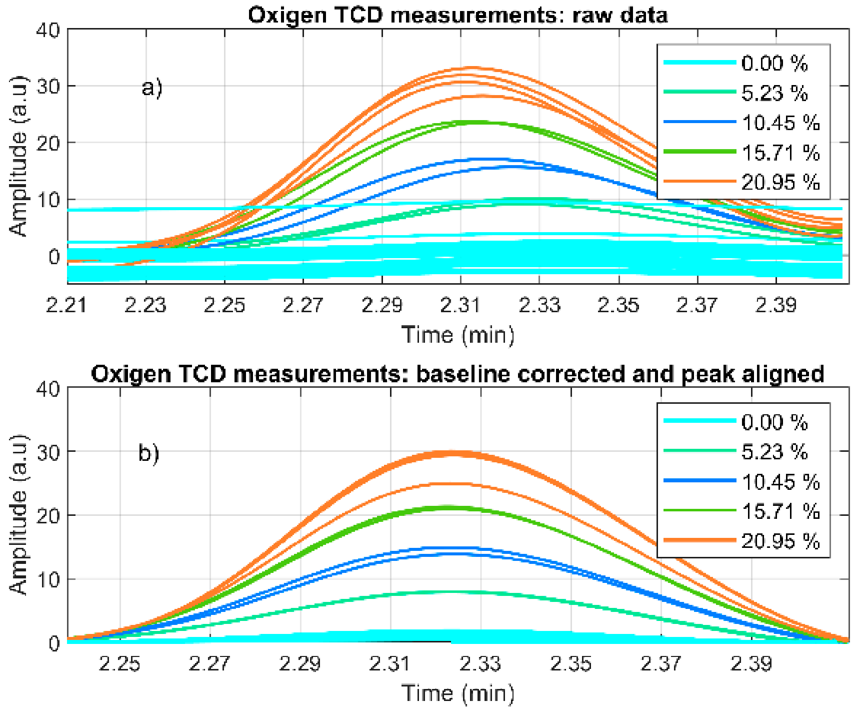

(a) Raw Oxygen TCD measurements. A misalignment of the peaks and different baseline levels can be observed

(b) Oxygen TCD measurements after baseline correction and peak alignment

Figure S3: Baseline correction and alignment for Nitrogen TCD measurement

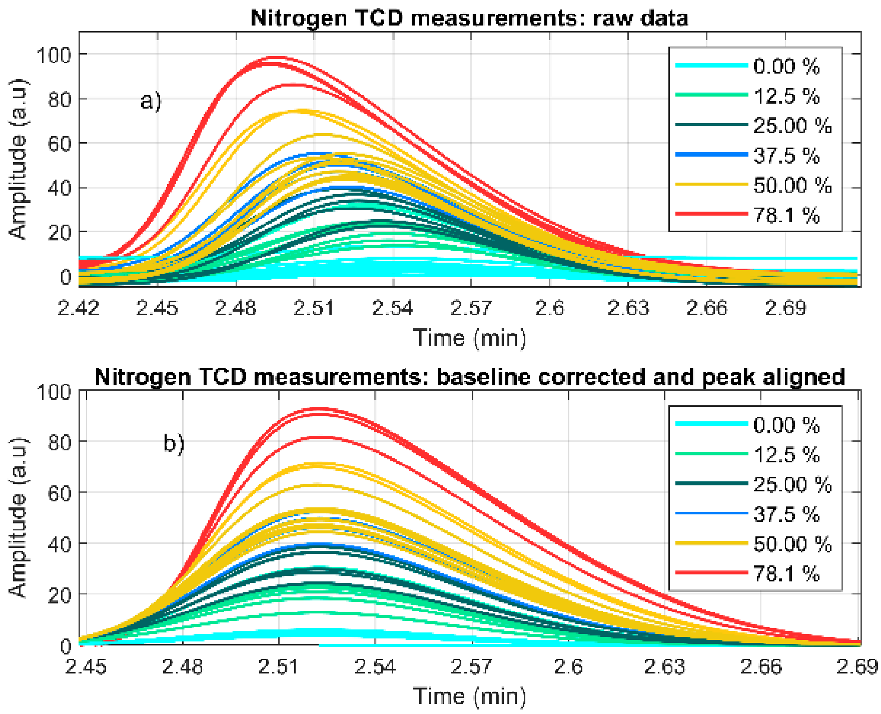

(a) Raw Nitrogen TCD measurements. A misalignment of the peaks and different baseline levels can be observed;

(b) Nitrogen TCD measurements after baseline correction and peak alignment

Figure S4: Baseline correction and alignment for Methane TCD measurement

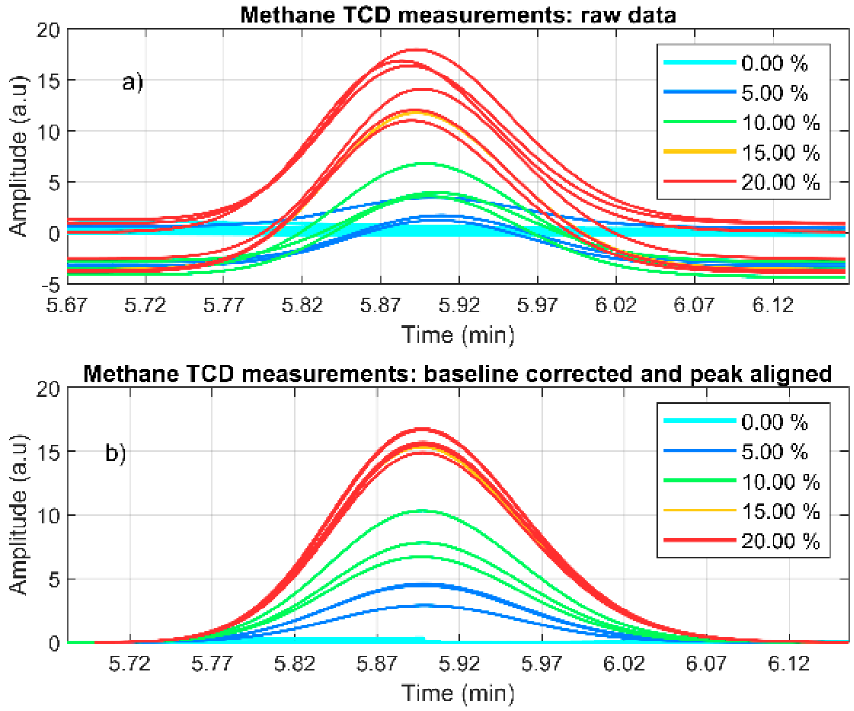

(a) Raw Methane TCD measurements. A misalignment of the peaks and different baseline levels can be observed;

(b) Methane TCD measurements after baseline correction and peak alignment

Table S1: Healthy subjects submitted to low flatulogenic diet and high flatulogenic diet. The 5 major gases concentration is reported jointly the 95% confidence interval. Also, some biometric parameters are added: age, sex, and IMC of every subject. A total amount of 20 measures has been performed

Sex	Age	IMC	Vol. (ml)	Diet		H_2	O_2	N2	CH4	CO2
Female	32	21.3	43	Fibre	Measure 1 (%)	0.0	36.2	34.7	20.7	8.56
					Conf. Inter.(%)	-0.5, 0.5	35.6, 37.5	30.3, 39,0	20.5, 22.0	7.7, 8.8
					Measure 2 (%)	0.0	36,7	34.9	20.2	8.2
					Conf. Inter.(%)	-0.5, 0.5	36,2, 38,1	30.5, 39,2	20.0, 21,5	7,4, 8.5
			34	Protein	Measure 1 (%)	1.4	6.5	65.4	22.1	4.6
					Conf. Inter.(%)	0.8, 1.9	6.2, 6.9	59.1, 71.6	21.9, 23.5	4.0, 4.9
					Measure 2 (%)	1.4	6.91	65.3	21.8	4.5
					Conf. Inter.(%)	0.85, 2,0	6.7, 7.3	50.1, 71.2	21.6, 23.1	3.9, 4.8
Male	31	24.7	555	Fibre	Measure 1 (%)	0.5	7.8	31.4	44.1	16.3
					Conf. Inter.(%)	-0.0, 1,1	7.5, 8.2	27.2, 35.5	43.8, 46.5	14.9, 16.5
					Measure 2 (%)	0.5	8.0	31.4	43.9	16.2
					Conf. Inter.(%)	-0.1, 1.0	7.7, 8.4	27.3, 35.5	43.7, 46.4	14.8, 16.5
			380	Protein	Measure 1 (%)	0.0	0.6	51.6	40.6	7.5
					Conf. Inter.(%)	-0.5, 0.5	0.4, 0.8	46.2, 57.0	40.5, 42.8	6.7, 7.7
					Measure 2 (%)	0.0	0.6	51.4	40.5	7.5
					Conf. Inter.(%)	-0.5, 0.5	0.4, 0.8	46,0, 56,8	40.4, 42.8	6.3, 7.8
Male	30	26.1	780	Fibre	Measure 1 (%)	0.0	1.0	46.1	15.6	37.5
					Conf. Inter.(%)	-0.5, 0.5	0.9, 1.2	41.0, 51.1	15.4, 16.6	34.7, 37.7
					Measure 2 (%)	0.0	0.2	44.9	16.3	39.0
					Conf. Inter.(%)	-0.5, 0.5	0.0, 0.4	40.0, 49.9	16.0, 17.3	36.1, 39.3
			439	Protein	Measure 1 (%)	0.0	0.1	57.3	24.6	18.2
					Conf. Inter.(%)	-0.5, 0.5	-0.0, 0.3	51.5, 63.0	24.4, 26.1	16.7, 18.4
					Measure 2 (%)	0.0	0.0	55.5	26.1	19.3
					Conf. Inter.(%)	-0.5, 0.5	-0.2, 0.2	49.8, 61.1	25.9, 27.7	17.7, 19.6
Female	29	23.5	148	Fibre	Measure 1 (%)	3.0	1.6	48.6	37.2	9.5
					Conf. Inter.(%)	2.4, 3.6	1.4, 1.8	43.4, 53.8	37,0, 39.3	8.6, 9.8
					Measure 2 (%)	2.7	1.9	49.5	36.6	9.3
					Conf. Inter.(%)	2.0, 3.3	1.7, 2.2	44.2, 54.8	36.4, 38.6	8.4, 9.6
			82	Protein	Measure 1 (%)	0.0	7.2	51.3	33.6	8.2
					Conf. Inter.(%)	-0.5, 0.5	6.9, 7.6	45.9, 56.6	33.4, 35.6	7.3, 8.5
					Measure 2 (%)	0.0	7.0	51.5	33.2	8.3
					Conf. Inter.(%)	-0.5, 0.5	6.7, 7.4	46.2, 56.9	33.0, 35.1	7.5, 8.6
Female	36	18.6	92	Fibre	Measure 1 (%)	2.1	0.0	57.7	26.5	12.5
					Conf. Inter.(%)	1.5, 2.7	-0.2, 0.2	52.0, 63.5	26.3, 28.1	11.4, 12.8
					Measure 2 (%)	1.9	0.3	56.3	27.9	13.3
					Conf. Inter.(%)	1.3, 2.5	0.2, 0.5	50.1, 62.1	27.7, 29.6	12.2, 13.6
			40	Protein	Measure 1 (%)	0.8	2.3	64.8	22.9	9.2
					Conf. Inter.(%)	0.2, 1.3	2.1, 2.6	58.6, 71,0	22,6, 24,3	8.3, 9.5
					Measure 2 (%)	0.9	3.0	65.2	22.2	8.8
					Conf. Inter.(%)	0.3, 1.4	2.8, 3.3	58.9, 71.4	21.9, 23.5	7.9, 9.1
